# Supplementary material for: Potential sources of contamination on textiles and hard surfaces identified as high-touch sites near the patient environment
Source: PLoS One. 2023 Jul 7;18(7):e0287855. doi: 10.1371/journal.pone.0287855 (PMC10328241; doi:10.1371/journal.pone.0287855)
Supplement: S1 Table — (PDF) [file pone.0287855.s001.pdf]

S1 Table. Description of the 26 samples analysed by 16S rDNA sequencing

Accession number	Category	Surface
SAMN32777245	Textile	Babynest
SAMN32777246	Textile	Clothing
SAMN32777247	Textile	Clothing
SAMN32777248	Textile	Babynest
SAMN32777249	Hard surface	Table
SAMN32777250	Hard surface	Table
SAMN32777251	Hard surface	Around bed
SAMN32777252	Hard surface	Device
SAMN32777253	Hard surface	Device
SAMN32777254	Hard surface	Device
SAMN32777255	Hard surface	Bathroom
SAMN32777256	Hard surface	Bathroom
SAMN32777257	Textile	Furniture
SAMN32777258	Textile	Furniture
SAMN32777259	Textile	Furniture
SAMN32777260	Textile	Furniture
SAMN32777261	Textile	Sheet
SAMN32777262	Hard surface	Bathroom
SAMN32777263	Hard surface	Mop
SAMN32777264	Hard surface	Mop
SAMN32777265	Textile	Clothing
SAMN32777266	Hard surface	Device
SAMN32777267	Hard surface	Table
SAMN32777288	Hard surface	Table
SAMN32777299	Hard surface	Table
SAMN32777270	Hard surface	Mop
