# Supplementary material for: Potential sources of contamination on textiles and hard surfaces identified as high-touch sites near the patient environment
Source: PLoS One. 2023 Jul 7;18(7):e0287855. doi: 10.1371/journal.pone.0287855 (PMC10328241; doi:10.1371/journal.pone.0287855)
Supplement: Attachment — Submitted filename: PLOS one review_Apr 11, 23.docx [file pone.0287855.s002.docx]

PLOS one review

Potential sources of contamination on textiles and hard surfaces identified as high-
touch sites near the patient environment

Introduction

Line 48:

Even though, not Even tough

Methods

Comment: **The method section can be enhanced by breaking some of the subsections further. For instance, the first subsection ‘**Study environment and observational study (now study design)’ can be divided into 2 separate sections.

Observational study is too broad, and it comprises several study designs such as case reports, case series, cross-sectional study, etc (<https://www.ncbi.nlm.nih.gov/pmc/articles/PMC6003013/>). I suggest that the authors be specific with their study design, and replace all observational study with the specific design.

Line 87:

‘Study environment and study design’ is more appropriate

Line 89:

Replace ‘during’ with ‘from’

Line 101 – 105:

I suggest this paragraph stand alone as a subsection titled ‘Ethical Approval’. Also, I suggest you provide approval number from the committee.

Line 107 – 141:

As mentioned previously, break this subsection further. So, the authors can talk about sample collection alone, then sample preparation, etc…

Not antibiotic susceptibility tests performed???

Line 123:

Rinsing with what? Just water, or soapy water??

Line 168:

I suggest significant value be p< 0.05, and not p=0.05.

Results

Line 172 – 176:

This paragraph is not necessary under results. The authors should only talk about the results obtained after they carried out their experiment, and not to give explanations or preambles.

So, from the results, there was no *C. difficile* isolated and identified during the culture phase. This should be stated clearly.

Table 1:

I suggest the authors define the values inside the bracket/parenthesis. Since n=176 for both columns, you can include the ‘n=176’ at the end of the table 1 title. Then, you can define the column as n(%), meaning you have the values on the outside and their respective percentages inside the parenthesis. Don’t mixed the decimal points up (be consistent with the ‘,’ or ‘.’).

Table 2:

Wondering why the authors didn’t identify further to the species level. We know not all *Staphylococcus, Strep, Pseudo* etc are normal flora. So, speciation would have given us more insight into which category of organisms we are dealing with: whether pathogenic types or not.

Line 206:

In addition to, not ‘in addition~~al~~ to’

Line 335:

Lack of evidence. Insert ‘of’.

The authors should let us know if the study had any limitation(s). This can be part of the discussion.

**General comments:**

1. I know multidrug resistance is a big problem in hospital settings. Why didn’t the authors perform antibiotic susceptibility tests on the isolated microorganisms?

**2. I suggest authors get a native English speaker to proof-read the entire work and make necessary corrections to all erroneous spellings, punctuations and grammatical expressions.**
